# Vitamin D deficiency in patients with Behcet’s disease

**DOI:** 10.1186/2251-6581-13-18

**Published:** 2014-01-22

**Authors:** Seyedeh Tahereh Faezi, Narges Ansari, Pedram Paragomi, Maassoumeh Akhlaghi, Majid Ghanavat, Fereydoun Davatchi

**Affiliations:** 1 Rheumatolology Research Center (RRC), Shariati Hospital, Tehran University of Medical Sciences,(TUMS), Kargar Avenue, Tehran, Iran

**Keywords:** Behcet’s disease, Internal medicine, Vitamin D

## Abstract

**Background:**

Behcet’s disease is an autoimmune, recurrent and multisystem disease. Vitamin D has immunomodulator role in immune system. So that vitamin D deficiency was reported in some autoimmune diseases. Behcet’s disease as a Silk Road disease is common in Iran. The aim of this study was to detect the serum level of 25(OH) vitamin D in Behcet’s patients and control group.

**Methods:**

In this case–control study, 112 Behcet’s patients as cases group and 112 healthy individuals as controls group were enrolled. Any subject on vitamin D supplement, steroid, and immunosuppressors during the last 6 months were excluded. The serum level of 25(OH) vitamin D was measured in the two groups by ELISA method. The findings were compared via SPSS software.

**Results:**

About 57% and 17% of Behcet’s patients had vitamin D deficiency and insufficiency respectively. Vitamin D deficiency was significantly more common in controls than cases group (P < 0.001). Vitamin D levels were significantly lower in controls (P < 0.001). Age and sex did not have any confounding effect on the results. There was no significant relationship between disease duration, disease activity, Pathergy test, HLA-B5, and HLA-B51 with vitamin D level in Behcet’s patients.

**Conclusions:**

Vitamin D deficiency is common among Behcet’s patients. However, our results revealed vitamin D deficiency was significantly more common in healthy controls in comparison with Behcet’s cases.

## Introduction

Behcet’s disease (BD) is a chronic relapsing disease of autoimmune nature which involves multiple systems [1,2]. One of the characteristics of this disease is its mucocutaneous lesions (oral or genital aphthosis), major organ involvements such as ocular, CNS or vascular manifestations [3,4]. The disease is known for its ethnic basis, spreading through ancient Silk Road [5,6]. Iran is the second most prominent foci for BD with prevalence rate of 80 out of 100,000 [7]. The ethiopathogenesis of Behcet’s disease is not fully understood. However, genetical, environmental and immunological factors contribute to disease progression [8-10]. Immune mechanisms play a major role in Behcet’s disease [11]. Heat shock proteins, cytokines, alterations in neutrophil and macrophage activity, and autoimmune mechanisms have all been implicated [12-15]. Circulating immune complexes also play a role in precipitating the characteristic neutrophilic vascular reaction [16].

Active vitamin D (Vit-D), or 1,25(OH) D3 has been linked with the metabolism of calcium and phosphorus [11]. Moreover, studies have unraveled the role of vitamin D in the regulation of immune responses [12]. Known roles of vitamin D in immune system include inhibiting proliferation of T Helper_1_ cells [13,14], stimulation of regulatory T-cells [15,16], diminishing B-lymphocyte differentiation and inhibiting immunoglobulin secretion [17]. In addition, restricted antigen presentation via macrophages and modulation of dendritic cells maturation have been linked with vitamin D [18]. Previous studies have divulged lower levels of 25(OH) Vitamin D in autoimmune diseases such as Diabetes Mellitus, Systemic lupus erythematosus (SLE), Multiple sclerosis, inflammatory bowel disease and rheumatoid arthritis (RA), compared with healthy individuals [19-22]. Due to high prevalence of Behcet’s disease in Iran and the important role of vitamin D in the autoimmune responses, we conducted this study to evaluate vitamin D level in BD patients and investigate whether a difference in vitamin D level exist between BD cases and healthy controls.

## Patients and methods

The applied protocol in the current research project has been approved by Ethical Board Committee of the Rheumatology research center. Patients group comprised of cases who were referred to Behcet’s clinic and diagnosed with Behcet’s disease according to international study group criteria (ISG) for BD. Exclusion criteria for BD subgroup consisted of history of taking any kind of vitamin D supplements (pearl, injection, Multivitamin or Ca-D), any kind of corticosteroids or cytotoxic drugs during the last 6 months. Control group, included healthy volunteers from the staff of Shariati hospital. Individuals with history of chronic disease including renal or hepatic diseases, bone metabolic disease, malabsorption, type 1 diabetes mellitus or malignancies were excluded from study. Furthermore controls with history of vitamin D, Ca-D, Multivitamin or any drug affecting bone metabolism (such as phenobarbital or phenytoin) in the last 6 months were excluded. Patients and controls were ascribed consecutively after explanation of study plot. Patients who agreed on the terms of the study were asked to sign a written informed consent form. Thereafter patients were introduced to the lab of the Endocrinology and Metabolism Research Institute (EMRI) of Tehran University of Medical Sciences. Vitamin D measurements were performed via ELISA method. Vitamin D levels lower than 20 ng/ml were ascribed to “vitamin D deficiency” category. Vitamin D levels higher than 20 ng/ml and lower than 32 ng/ml were ascribed to “vitamin D insufficiency” and higher than 32 ng/ml of vitamin D were categorized as “Normal”. Patients’ characteristics including demographic features, disease duration, and clinical manifestations were registered through patients’ files and Behcet’s clinic registry. Furthermore, we utilized a tailored questionnaire for each patient to detail patients’ therapeutic and follow up information. The Rheumatologists of Behcet’s clinic exclusively assessed disease activity in each patient. According to the Physician Global Assessment (PGA) BD was divided to active and silent statuses. Active status was subdivided into three subgroups of “mild”, “moderate” and “severe”. SPSS 20.0 software was utilized to analyze the extracted data and comparison between various subgroups.

## Results

A total number of 224 BD patients and healthy controls were studied. BD subgroup comprised of 112 cases (46 males and 66 females) with mean age of 40.5 years (range: 18–70). Controls comprised of 112 healthy individuals (22 males and females) with mean age of 37.5 years (range: 23–65). Among BD patients 57.1% had vitamin D deficiency and 16.9% had vitamin D insufficiency. Meanwhile in control group, 91.1% had Vit-D deficiency and 2.7% had vitamin D insufficiency. These findings indicate a significant difference between BD patients and controls in prevalence of vitamin D deficiency (P < 0.001). Average level of vitamin D in BD cases and healthy controls were 24.4 ± 2.5 and 11.1 ± 1.6 ng/ml respectively (P < 0.001) (Table 1).

**Table 1:** Serum vitamin D level in Behcet’s cases and controls group

**Vitamin D level**	**Sex**	**Deficiency number (%)**	**Insufficiency number (%)**	**Normal number (%)**	**Total number**
**BD patients**	**Male**	23(20.5%)	9(8.0%)	15(13.4%)	66
	**Female**	41(36.6%)	10(8.9%)	14(12.5%)	46
**Controls**	**Male**	22(19.6%)	0	0	22
	**Female**	80(71.4%)	3(2.7%)	7(6.2%)	90

Female individuals constitute 58.9% of BD cases and 80.4% of controls. Therefore a significant difference in gender distribution between BD and control subgroup existed (P < 0.001). In BD patients a significant difference between men and women was detected in vitamin D level (24.8 ± 3.9 ng/ml in women vs. 23.7 ± 2.4 ng/ml in men, P = 0.039). On the contrary, comparison of vitamin D levels between male and female individuals of the control group did not reveal a significant difference (12.1 ± 1.9 ng/ml in female controls vs. 6.9 ± 0.9 ng/ml in male controls, P = 0.064). Mean age of BD patients and controls were 40.5 ± 1.03 and 37.5 ± 0.7 respectively (P = 0.010). Our analysis revealed no significant correlation between age and vitamin D level (P = 0.055, r = 0.128).

Turkish ethnicity was significantly more common in BD cases in comparison with controls (43.8% vs. 24.1%, P = 0.002). In BD patients no significant difference in vitamin D level was detected between Turkish and Non-Turkish cases (31.02 ± 4.9 in Turkish and 19.2 ± 2.1 ng/ml in Non-Turkish patients, P = 0.055). Likewise, no significant difference in vitamin D levels in Turkish and Non-Turkish controls was detected (8.3 ± 1.1 vs. 11.9 ± 2.09 ng/ml, P = 0.114).

In BD group the mean time span from BD criteria fulfillment and established diagnosis to last patients’ visit to BD clinic was 7.98 ± 6.8 years. No significant correlation between diagnosis to last visit interval and vitamin D level was detected (P = 0.071, r = 0.171). Mean time interval between onset sign and last patient’s visit to BD clinic was 14.5 ± 8.8 years. No correlation between disease duration (time span from onset sign to last visit) and vitamin D level was detected (P = 0.071, r = 0.171).

Twenty-one BD patients (18.7%) had active disease while 91 (81.3%) were in remission. No correlation between disease activity and vitamin D levels was present (P = 0.145, r = 0.138). In 14 cases (12.5%) mild disease activity and in 7 cases (6.2%) moderate disease activity was noted. None of the studied cases had severe BD activity. Among BD patients, positive pathergy test was obtained in 69 cases. In this subpopulation mean vitamin D levels was 24.7 ± 3.4 ng/ml. On the other hand, in 31 BD patients who had negative pathergy test, mean vitamin D level was 25.5 ± 4.8 ng/ml. Vitamin D measurements did not reveal significant difference between these two subgroups (P = 0.772).

In BD patients, HLA-B51 was positive in 36 cases (40.9%) with mean vitamin D level of 19.8 ± 3.4 ng/ml and negative in 52 cases (59.1%) with mean vitamin D level of 24.2 ± 3.4 ng/ml. Statistical analysis revealed no significant difference between these two subgroups of BD (P = 0.248). Positive HLA-B5 was detected in 53 BD cases (53.5%), while 46 BD cases had negative HLA-B5 (46.5%). Mean vitamin D level in HLA-B5 positive patients was 25.1 ± 3.6 and in HLA-B5 negative cases it was 21.88 ± 3.4 ng/ml (P = 0.776).

## Discussion

Vitamin D deficiency is a global public health concern which affects various age groups [23]. Arabi *et al.* reported hypovitaminosis D is encountered in 33 to 50% of general population in Sub-Saharan Africa and Middle East [24]. Previous surveys in Middle East have pointed out the sun exposure, latitude, Islamic clothing, body mass index (BMI), Calcium supplements and genetic factors are major determinants of vitamin D level [25-28].

One of the essential attributes of vitamin D is the immunoregulatory role [29-31]. Therefore vitamin D deficiencies need to be comprehensively addressed in patients with immunologic aberrancies. The optimum serum level of vitamin D which facilitates efficient immune responses is not precisely determined. However it is believed to be lower than vitamin D required level in healthy bone metabolism [12]. Plethora of studies has unraveled the higher rate of vitamin D deficiency among patients with autoimmune diseases, adding that the condition is correlated with disease activity [12,30,32,33]. It has been postulated that vitamin D deficiency results in diminished regulator T- cells and shifts Th1/Th2 ratio toward Th1 [34]. These findings underline the modulatory effect of vitamin D on T cells and inflammatory mediators [34].

Previous studies have investigated the vitamin D status in Behcet’s disease [29,34,35]. A number of these studies have revealed lower levels of serum vitamin D in active BD in comparison with normal controls [29,35,36]. Kandi *et al.* detected lower levels of vitamin D in BD patients in comparison with controls group. However this difference was not significant [37]. On the contrary our study unraveled a significantly higher level of vitamin D in BD patients in comparison with controls. The controversy may be partly due to the inclusion criteria of current study which excluded BD cases with severe activity. Moreover patients and controls were gathered at different timeframes which might have affected the results. Blood samples of BD patients were collected in late summer and early autumn, while as for the controls, they were recruited in late autumn and early winter.

BD patients are exclusively consulted on issues such as life style, physical activity and nutrition and have a better insight about their general health status. We postulated that the tailored education of BD patients may attribute to the lower rate of vitamin D deficiency. On the other hand, the considerable prevalence of vitamin D deficiency and insufficiency in our control group may indicate the poor health education in general population as well as other parameters such as lack of sun exposure or nutrition.

A number of studies have unraveled the positive relation between BD activity and vitamin D deficiency [29,35,36]. Another study by Hamzaoui *et al.* revealed lower level of vitamin D in active BD cases in comparison with silent BD cases and control group. However these results did not reveal a significant correlation between disease activity and vitamin D level [34]. Likewise we did not detect a significant correlation between disease activity or severity and vitamin D level. It is noteworthy that BD cases with severe activity were excluded in accordance with the instructions of our study. This is due to timely administration of immunosupressors and corticosteroids in patients with severe BD which met exclusion criteria of our study. Therefore Behcet’s disease was silent in most of studied patients (80%) at the time of study. The remaining 20% of BD patients were mostly comprised of mildly-active patients.

Our results revealed high rate of Vitamin D deficiency in control group and the overall figures demonstrated very low levels of serum vitamin D in majority of studied control population. These findings are in accordance with previous studies in Tehran and Iran, which had revealed the magnitude of problem in Iran [25,26]. According to the nationwide survey 75% of Iranian women and 72% of Iranian men suffer from vitamin D deficiency [25]. The higher rate of hypovitaminosis D in control group of our study (91% had vitamin D deficiency, and 2.7% had vitamin D insufficiency) may be attributed to limited sun exposure in the season of controls group enrollment (autumn and winter) and the younger age. Hashemipour *et al.* reported mean serum vitamin D level significantly increased with age in Iranian women [26]. In the current study, mean age of controls group was significantly lower than BD patients. Moreover mean age of both BD and control groups were lower than previous nationwide surveys. Thus the age parameter may partially justify the higher prevalence of hypovitaminosis D in our control group in comparison with nationwide survey.

Another factor which must be addressed is the Islamic clothing in Iranian women which may be influential as the significant difference in gender distribution existed between control and BD groups. However when patients and controls of both genders were compared separately, higher rate of vitamin D deficiency in two genders was redetected in both control and BD groups.

Ethnic distribution varied between control and BD groups as Turk patients are the majority of BD population, whilst controls resembled the overall distribution of ethnicities in Iran. However, our data analysis detected no significant difference in vitamin D level between Turkish and non-Turkish participants in both control and BD groups.

In the current study 37% of BD patients had a history of corticosteroid administration. The analysis revealed no significant difference in vitamin D level between patients who had received corticosteroid and patients who had not. This may be due to discontinuation of corticosteroid during 6 month period prior to enrollment which was a part of inclusion criteria.

In comparison with studies on other rheumatologic conditions, vitamin D deficiency was more prevalent among our BD group in comparison with Iranian SLE and RA cases [38,39]. These findings stress the necessity of vitamin D control in BD patients.

In majority of BD cases (69%), pathergy test was positive; however, no significant difference between pathergy reaction positivity and vitamin D level was noted. Likewise no correlation between HLA-B5 and HLA-B51 immunoreaction and vitamin D level was detected. These findings may downplay role of genetic factors in hypovitaminosis D in BD cohort.

To our knowledge previous studies had not assessed the correlation between factors such as HLA-B5, HLA-B51, pathergy test and mean disease duration and vitamin D level in BD.

This study had a number of limitations. We could not evaluate the confounding impact of some factors such as nutrition, BMI or genetic predisposition. Our BD cases were referred to the Behect’s clinic from various regions of our country, while all controls were inhabitants in Tehran, the Iranian capital. We could not synchronically enroll patients and controls in a same season therefore a seasonal difference existed between two groups. More scrutinized studies in Iranian BD patients are warranted to investigate the link between specific features of BD and serum vitamin D level.

## Conclusion

Our study stressed the high prevalence of vitamin D deficiency in BD population. However, in comparison with normal population; BD patients had significantly higher level of serum vitamin D which may be partly due to insightful education they obtain in Behcet’s clinc. Moreover this study did not detect any significant correlation between BD activity, HLA-B5, HLA-B51 and vitamin D level. However, more scrutinized studies are warranted to address the issue of vitamin D deficiency in BD and its relation with disease activity.

## Competing interest

The authors declare that they have no competing interests.

## Authors’ contributions

STF participated in sequence alignment and designed the study. NA Conducted the study and collected the data. PP participated in the sequence alignment, drafted the manuscript and performed the statistical analysis. MA participated in designation of study. MG participated in data collection and drafting the manuscript. FD supervised the project and scientifically revised the manuscript. All authors read and approved the final manuscript.
